# Supplementary material for: Multifocal, multiphenotypic tumours arising from an MTOR mutation acquired in early embryogenesis
Source: Oncogene. 2024 Sep 13;43(44):3268–76. doi: 10.1038/s41388-024-03137-7 (PMC11518995; doi:10.1038/s41388-024-03137-7)
Supplement: Supplementary file 1 — Supplementary Figures [file 41388_2024_3137_MOESM1_ESM.pdf]

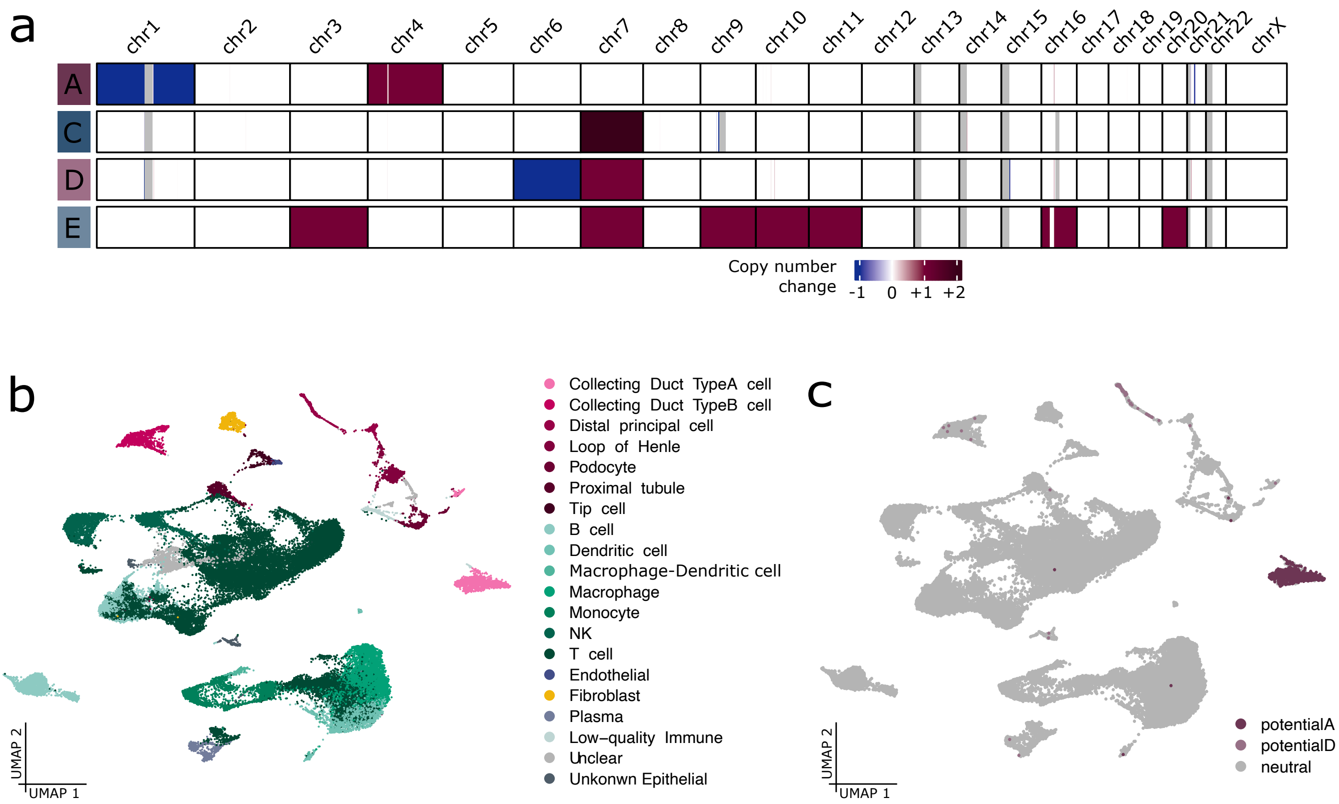
a
chr1
chr2
chr3
chr4
chr5
chr6
chr7
chr8
chr9
chr10
chr11
chr12
chr13
chr14
chr15
chr16
chr17
chr18
chr19
chr20
chr21
chr22
chrX
A
C
D
E
Copy number change
-1
0
+1
+2
b
Collecting Duct TypeA cell
Collecting Duct TypeB cell
Distal principal cell
Loop of Henle
Podocyte
Proximal tubule
Tip cell
B cell
Dendritic cell
Macrophage-Dendritic cell
Macrophage
Monocyte
NK
T cell
Endothelial
Fibroblast
Plasma
Low-quality Immune
Unclear
Unkonwn Epithelial
UMAP 2
UMAP 1
c
potentialA
potentialD
neutral
UMAP 2
UMAP 1

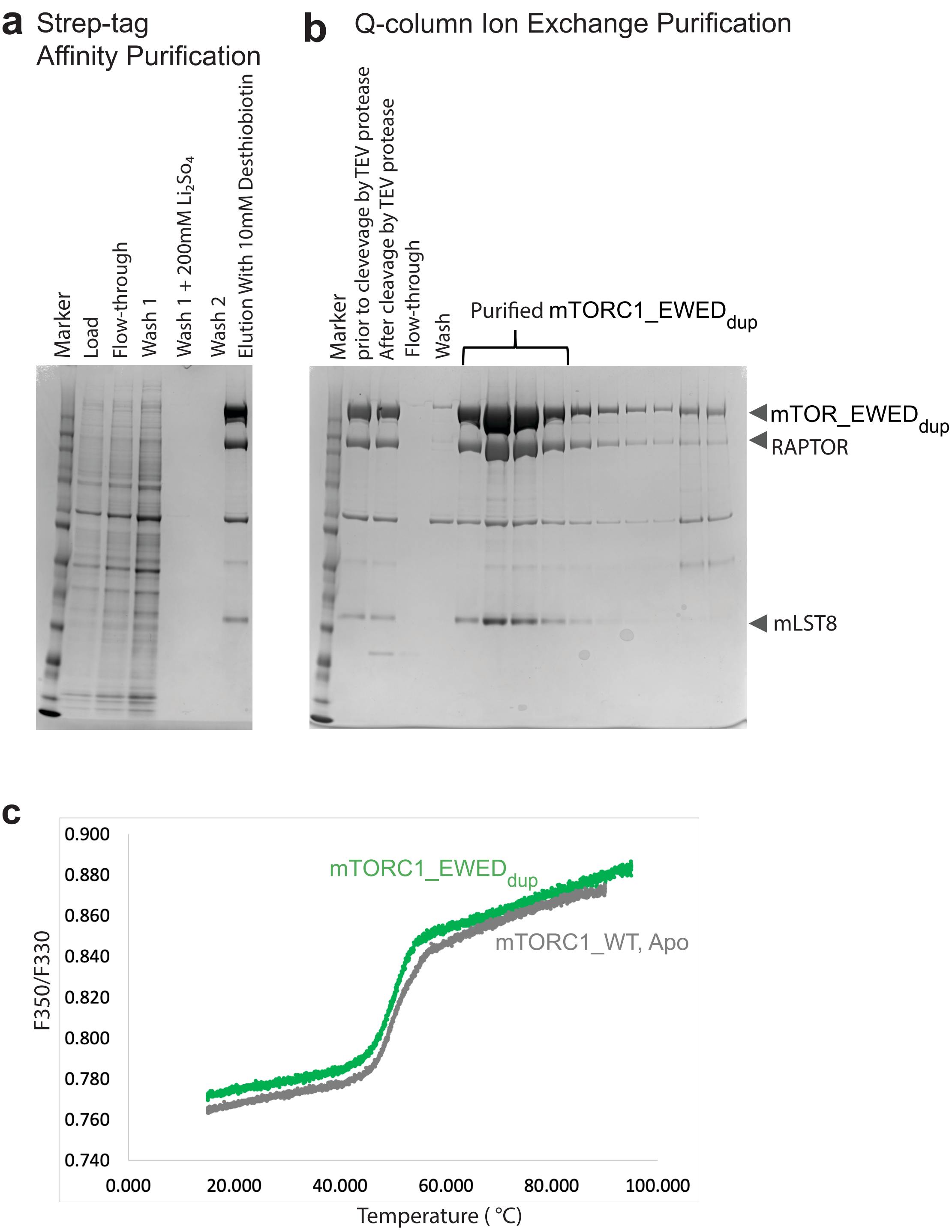

	Thermal Melting point (°C)	SD
mTORC1_WT, Apo	50.25	0.07
mTORC1_EWED$_{dup}$	50.05	0.05

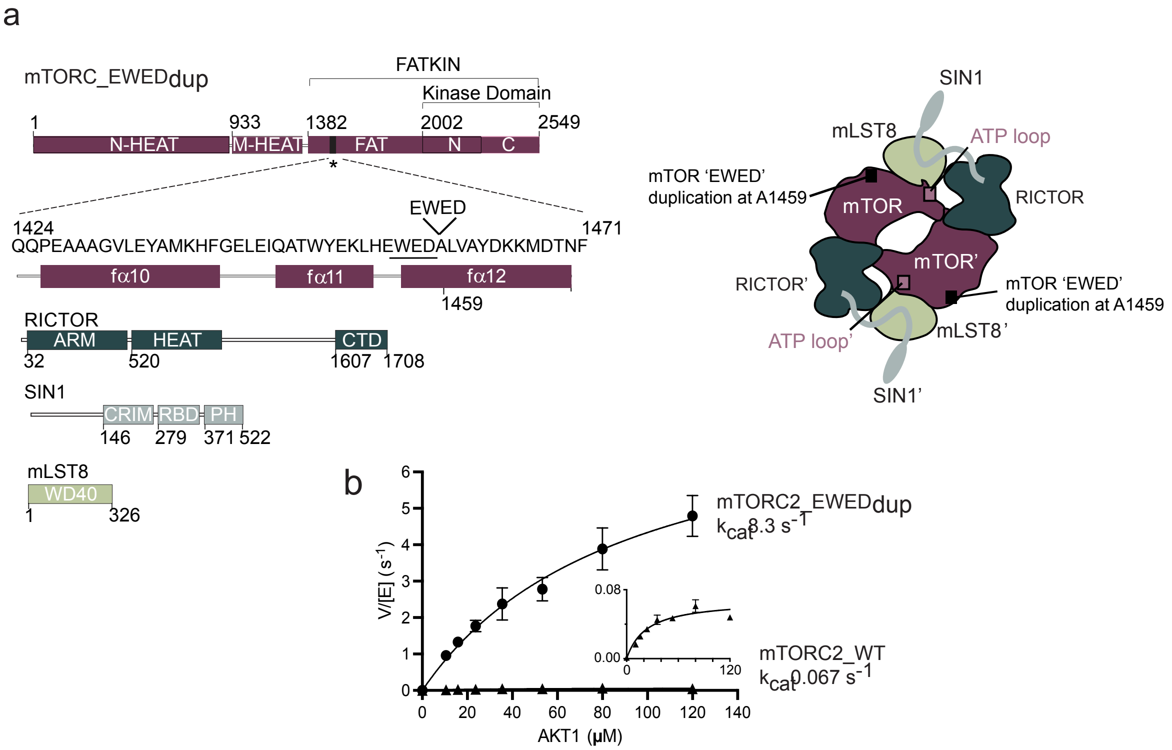
a
mTORC_EWEDdup
FATKIN
Kinase Domain
1
933
1382
2002
2549
N-HEAT
M-HEAT
FAT
N
C
*
EWED
1424
1471
QQPEAAAGVLEYAMKHFGELEIQATWYEKLHEWEDALVAYDKKMDTNF
fα10
fα11
fα12
1459
RICTOR
ARM
HEAT
CTD
32
520
1607
1708
SIN1
CRIM
RBD
PH
146
279
371
522
mLST8
WD40
1
326
SIN1
mLST8
ATP loop
mTOR 'EWED' duplication at A1459
mTOR
RICTOR
mTOR'
RICTOR'
mTOR 'EWED' duplication at A1459
mLST8'
ATP loop'
SIN1'
b
V/[E] (s-1)
AKT1 (μM)
mTORC2_EWEDdup
kcat 8.3 s-1
mTORC2_WT
kcat 0.067 s-1
0.08
0.00
0
120
0
1
2
3
4
5
6
0
20
40
60
80
100
120
140

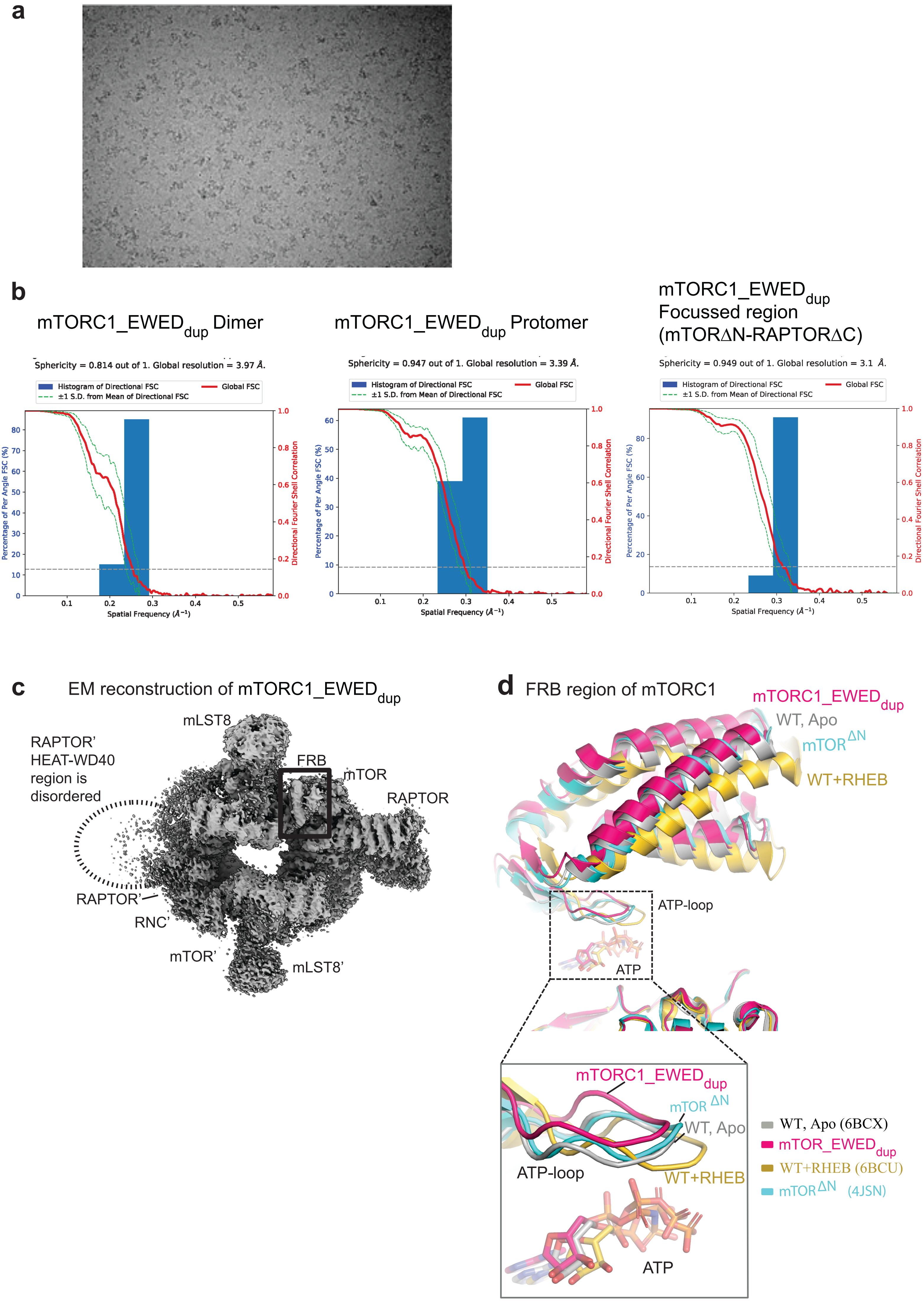
a
b
mTORC1_EWEDdup Dimer
Sphericity = 0.814 out of 1. Global resolution = 3.97 Å.
Histogram of Directional FSC
Global FSC
±1 S.D. from Mean of Directional FSC
Percentage of Per Angle FSC (%)
Directional Fourier Shell Correlation
Spatial Frequency (Å⁻¹)
mTORC1_EWEDdup Protomer
Sphericity = 0.947 out of 1. Global resolution = 3.39 Å.
mTORC1_EWEDdup
Focussed region
(mTORΔN-RAPTORΔC)
Sphericity = 0.949 out of 1. Global resolution = 3.1 Å.
c
EM reconstruction of mTORC1_EWEDdup
mLST8
RAPTOR' HEAT-WD40 region is disordered
FRB
mTOR
RAPTOR
RAPTOR'
RNC'
mTOR'
mLST8'
d
FRB region of mTORC1
mTORC1_EWEDdup
WT, Apo
mTOR ΔN
WT+RHEB
ATP-loop
ATP
WT, Apo (6BCX)
mTOR_EWEDdup
WT+RHEB (6BCU)
mTOR ΔN (4JSN)

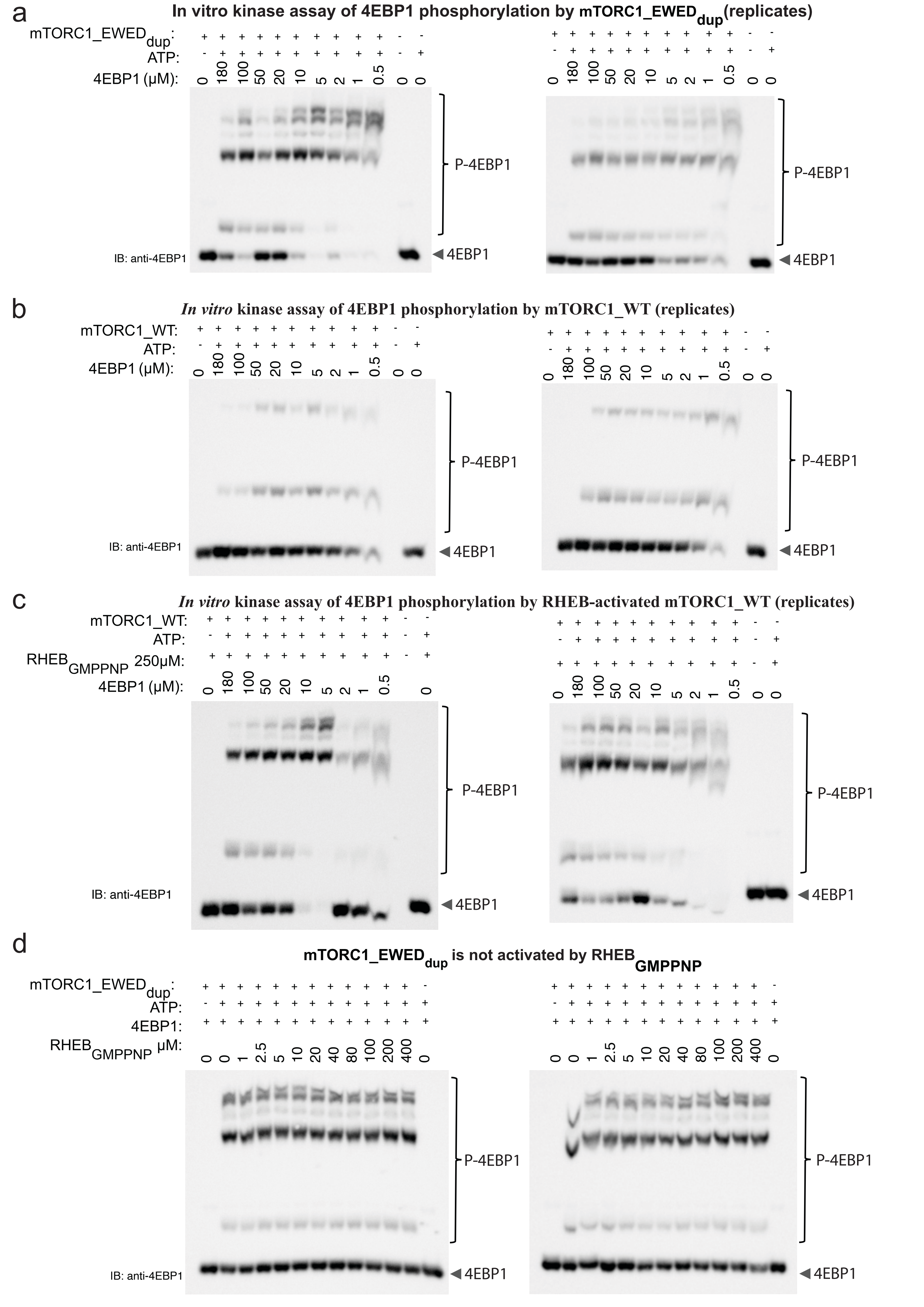

a
In vitro kinase assay of 4EBP1 phosphorylation by mTORC1_EWED$_{dup}$(replicates)
mTORC1_EWED$_{dup}$:
ATP:
4EBP1 (μM):
0 180 100 50 20 10 5 2 1 0.5 0 0
P-4EBP1
IB: anti-4EBP1
4EBP1
b
In vitro kinase assay of 4EBP1 phosphorylation by mTORC1_WT (replicates)
mTORC1_WT:
ATP:
4EBP1 (μM):
0 180 100 50 20 10 5 2 1 0.5 0 0
P-4EBP1
IB: anti-4EBP1
4EBP1
c
In vitro kinase assay of 4EBP1 phosphorylation by RHEB-activated mTORC1_WT (replicates)
mTORC1_WT:
ATP:
RHEB$_{GMPPNP}$ 250μM:
4EBP1 (μM):
0 180 100 50 20 10 5 2 1 0.5 0
0 180 100 50 20 10 5 2 1 0.5 0 0
P-4EBP1
IB: anti-4EBP1
4EBP1
d
mTORC1_EWED$_{dup}$ is not activated by RHEB$_{GMPPNP}$
mTORC1_EWED$_{dup}$:
ATP:
4EBP1:
RHEB$_{GMPPNP}$ μM:
0 0 1 2.5 5 10 20 40 80 100 200 400 0
P-4EBP1
IB: anti-4EBP1
4EBP1

In vitro kinase assays of AKT1_D274A phosphorylation by mTORC2_WT and mTORC2_EWED$_{dup}$

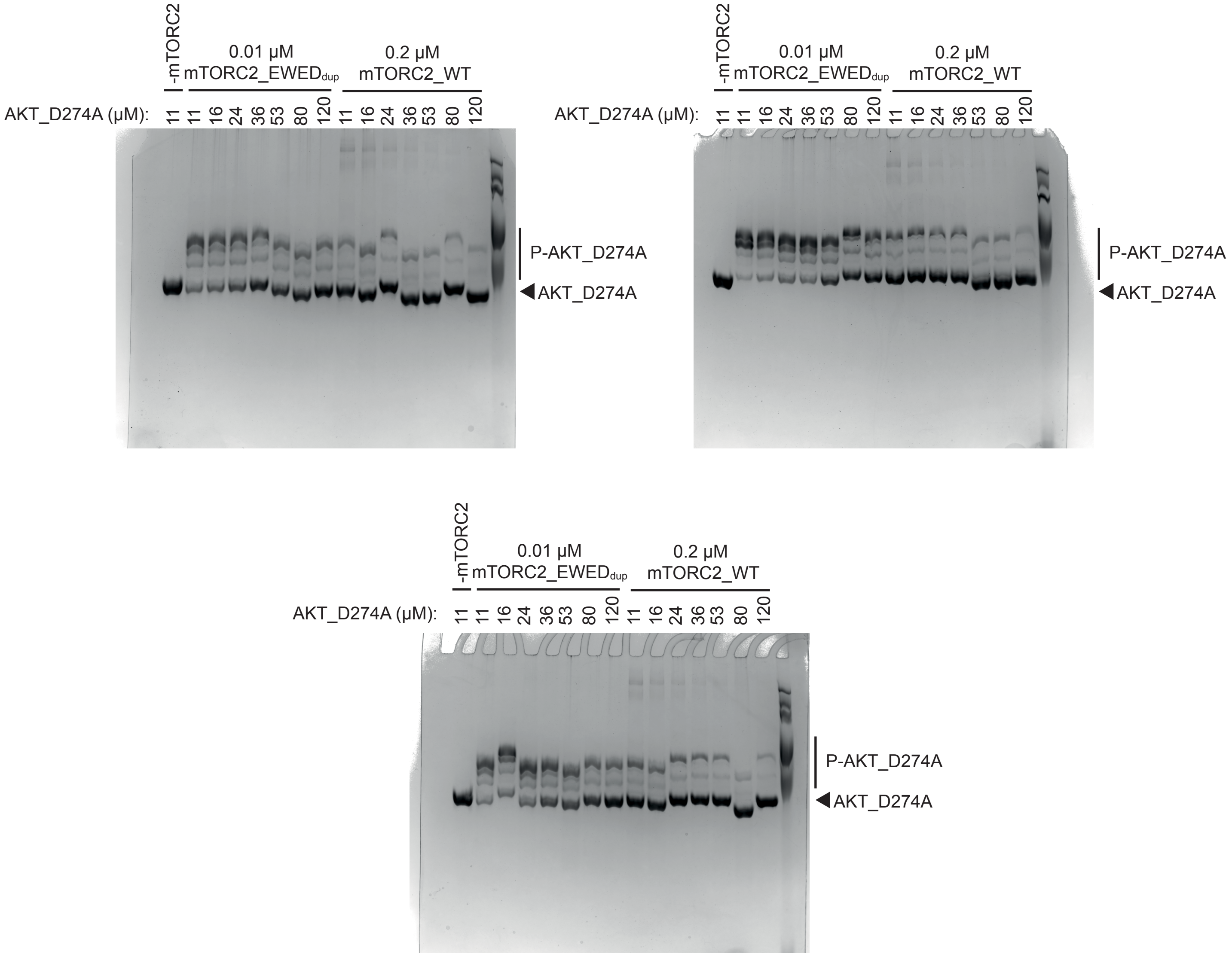

Source data for Extended Data Fig. 3b
