# Supplementary material for: Multifocal, multiphenotypic tumours arising from an MTOR mutation acquired in early embryogenesis
Source: Oncogene. 2024 Sep 13;43(44):3268–76. doi: 10.1038/s41388-024-03137-7 (PMC11518995; doi:10.1038/s41388-024-03137-7)
Supplement: Supplementary file 2 — Supplementary legends [file 41388_2024_3137_MOESM2_ESM.docx]

**Supplementary Figure Legends**

**Supplementary Fig. 1: Tumor aneuploidy and transcriptomic profiles.**

**(a)** Each tumor has a distinct copy number change profile.

**(b)** Cells are clustered and displayed in UMAP space, with each cluster labelled with a cell type identity inferred from logistic regression modelling.

**(c)** Cells harboring copy number changes that match oncocytomas A and D, highlighted in dark and light pink respectively, are grouped into two distinct clusters.

**Supplementary Fig. 2: mTORC1 purification and characterization.**

**(a,b)** SDS-PAGE showing the purification of mTORC1_EWED_dup_ variant. First step is strep-affinity purification **(a)**, followed by Ion Exchange Chromatography **(b)**.

**(c)** Thermal stability of mTORC1_WT and mTORC1_EWED_dup_ variant measured by differential scanning fluorimetry (DSF).

**Supplementary Fig. 3: mTOR_EWED_dup_ mutation hyperactivates mTORC2.**

**(a)** Schematic representation of mTORC2 subunits: mTOR, RICTOR, SIN1 and mLST8. The four-residue cancer-associated 1455-EWED-1458 duplication in the FAT region of mTOR is highlighted. Also shown is a schematic sketch of the subunit arrangement within a dimeric mTORC2 complex, with the regions of interest highlighted.

(**b**) Steady-state kinetic analysis of mTORC2_EWED_dup_ mutant phosphorylating full-length AKT1^ki^ (kinase-inactive mutant D274A). Inset: Zoomed in plot of mTORC2_WT to better visualize data. Reactions contained increasing concentrations of the AKT1 substrate as indicated, but an equal amount (1 µg) of total AKT1 from each reaction was loaded on a Phos-gel to achieve a linear range of detection. Gels were stained with Comassie InstantBlue stain and the intensities of the phosphorylated and nonphosphorylated bands were quantified with a ChemiDoc Touch Imaging System (Bio-Rad). Graphs show means ± SEM (with markers as indicated) of three independent experiments. Data are plotted as velocity over enzyme concentration.

**Supplementary Fig. 4: Cryo-EM structure determination of mTORC1_EWED_dup_ variant.**

**(a)** Top: A representative cryo-EM micrograph of mTORC1_EWED_dup_ variant.

**(b)** The FSC curves for the B-factor sharpened post-processed reconstructions suggest a final resolution of the 4.0 Å for mTORC1_EWED_dup_ Dimer (sphericity 0.8), 3.4 Å for mTORC1_EWED_dup_ protomer (sphericity 0.9) and 3.1 Å for mTORC1_EWED_dup_ focused protomer region (mTOR∆N-RAPTOR∆C, sphericity 0.9). The global resolution and the sphericity were calculated using 3D FSC server.

**(c)** Cryo-EM reconstruction of mTORC1_EWED_dup_.

**(d)** Alignment of cancer-associated mTORC1_EWED_dup_ structure (dark red) with apo mTORC1_WT (6BCX, gray), RHEB-activated mTORC1_WT (6BCU, orange) and the truncated mTOR_ΔN-HEAT_-mLST8 crystal structure (4JSN, cyan). The alignment was done on the kinase C-lobe of mTOR. The FRB region from the kinase N-lobe and the close view of the ATP-loop from all structures are shown, revealing that the mTORC1_EWED_dup_ hyperactivated mutant, similarly to the partially activated mTOR_ΔN-HEAT_-mLST8 truncation variant, does not adopt the stable signature features of the activated RHEB-mTORC1, such as the realignment of the ATP-binding loop in the N-lobe with the catalytic residues in the C-lobe.

**Source data for Fig. 3b: Phosphorylation of 4EBP1 by mTORC1_EWED_dup_ mutant in the absence and presence of RHEB-GMPPNP.**

(**a**) Immunoblots showing the phosphorylation of full-length 4EBP1 by mTORC1_EWED_dup_ mutant. Reactions contained increasing amount of the 4EBP1 substrate as indicated, but an equal amount (70 ng) of total 4EBP1 from each reaction was loaded on a Phos-gel in order to achieve a linear range of detection for quantitative Western blot analysis. Two independent experiments are shown.

(**b**) Immunoblots showing the phosphorylation of 4EBP1 by mTORC1_WT.

(**c**) Immunoblots showing the phosphorylation of 4EBP1 by mTORC1_WT in the presence of 250 μM RHEB-GMPPNP.

(**d**) Immunoblots showing the phosphorylation of 4EBP1 by mTORC1_EWED_dup_ mutant in the presence of increasing RHEB-GMPPNP concentration as indicated. The 4EBP1 was at a constant concentration of 10 μM. RHEB does not further activate mTORC1_EWED_dup_ to phosphorylate 4EBP1. A total amount of 70 ng of total 4EBP1 was loaded on a Phos-gel. Two independent experiments are shown.

**Source data for Supplementary Fig. 3b.**

Comassie InstantBlue-stained Phos-gel showing the phosphorylation of the AKT1 substrate by mTORC2_EWED_dup_ and mTORC2_WT, as indicated. Three independent experiments are shown.

**Supplementary Table 1:** Somatic SNVs in each sample as called with CaVEMan and filtered with beta binomial, shearwater-like, and fisher germline tests.

**Supplementary Table 2:** Variants shared by all four tumours with VAF adjustments according to copy number aberrations and purity.

**Supplementary Table 3:** ASCAT chromosome copy number results per sample.

**Supplementary Table 4:** Differential expression analysis - positive log2FoldChange corresponds to enrichment in tumours as compared to GTEx normal.

**Supplementary Table 5:** Gene set enrichment analysis with hallmark pathways - positive NES corresponds to enrichment in tumours as compared to GTEx normal.

**Supplementary Table 6:** Cryo-EM data collection, refinement, and validation statistics.
